# Supplementary material for: Functional analysis of recurrent CDC20 promoter variants in human melanoma
Source: Commun Biol. 2023 Nov 29;6:1216. doi: 10.1038/s42003-023-05526-2 (PMC10686982; doi:10.1038/s42003-023-05526-2)
Supplement: Supplementary file 2 — Supplementary Information [file 42003_2023_5526_MOESM2_ESM.pdf]

Supplementary Information

a

Name	Motif	p-value
Selected Motifs from distict families in pMRRs		
CTCF(Zf)/CD4+-CTCF-ChIP-Seq (Barski_et_al.)/Homer	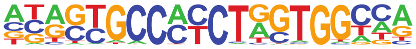	1e-6092
Fra1(bZIP)/BT549-Fra1-ChIP-Seq (GSE46166)/Homer	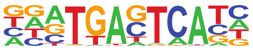	1e-4839
Sox10(HMG)/SciaticNerve-Sox3-ChIP-Seq (GSE35132)/Homer	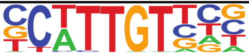	1e-472
Top 3 Motifs in Significant Hotspots		
Elk1(ETS)/Hela-Elk1-ChIP-Seq (GSE31477)/Homer	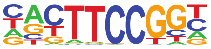	1e-178
Elk4(ETS)/Hela-Elk4-ChIP-Seq (GSE31477)/Homer	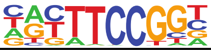	1e-164
ELF1(ETS)/Jurkat-ELF1-ChIP-Seq (SRA014231)/Homer	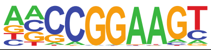	1e-161
Top 3 Motifs in 20 bp WT Sequence		
Elk1(ETS)/Hela-Elk1-ChIP-Seq (GSE31477)/Homer	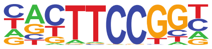	1e-311
ELF1(ETS)/Jurkat-ELF1-ChIP-Seq (SRA014231)/Homer	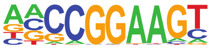	1e-260
Elk4(ETS)/Hela-Elk4-ChIP-Seq (GSE31477)/Homer	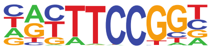	1e-145
Motif in 20 bp Mutated Sequence		
Ik-1	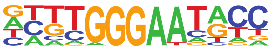	1e-10

b

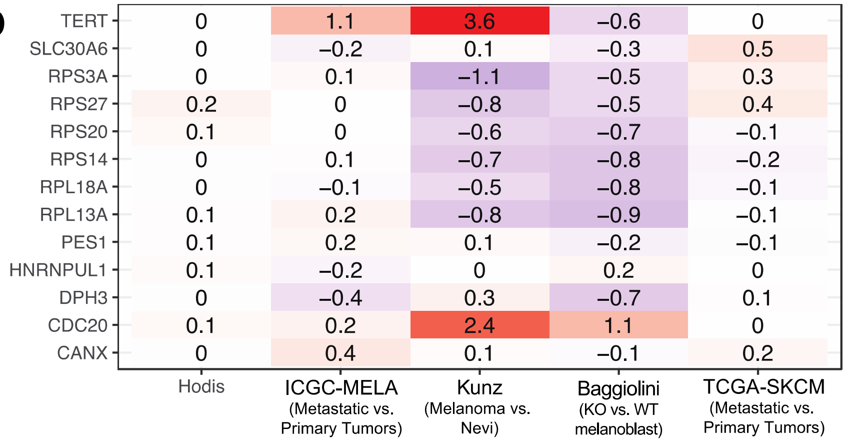

c

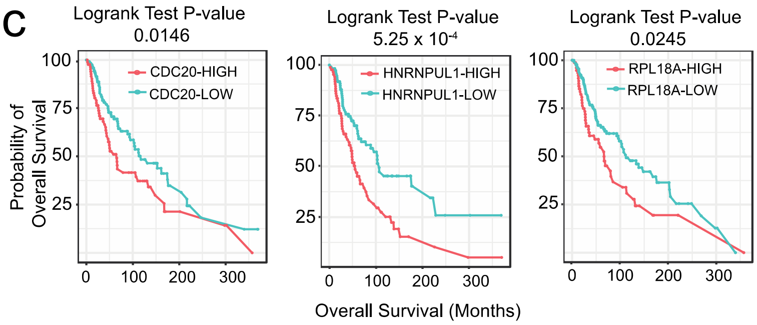

Supplemental Figure 1. Characterization of putative melanoma regulatory regions, hotspots, and associated genes. (a) Table of selected motifs identified by Homer analysis. First section shows results for all pMRRs, regardless of whether region harbors a hotspot. To showcase diversity of transcription factors, we chose high-ranking motifs from three distinct transcriptional families. Second section shows top 3 motifs for pMRRs harboring statistically significant hotspots (707 hotspots, FDR-adjusted p-value < 0.05). Last two sections show top 3 motifs when input is a 20 bp sequence containing either the WT (top) or mutant (bottom) allele for all variants within statistically significant hotspots. **(b)** Log_2 Fold-Change for Top 13 genes in ICGC-MELA, TCGA-SKCM, Kunz, and Baggiolini. Order in which samples are written represents numerator and denominator (e.g. if higher in metastatic, positive fold-change). **(c)** Kaplan-Meier curves representing overall survival rates for high (red) and low (blue) expressing tumors for the three genes listed. Data and p-values obtained from cBioPortal using OQL.

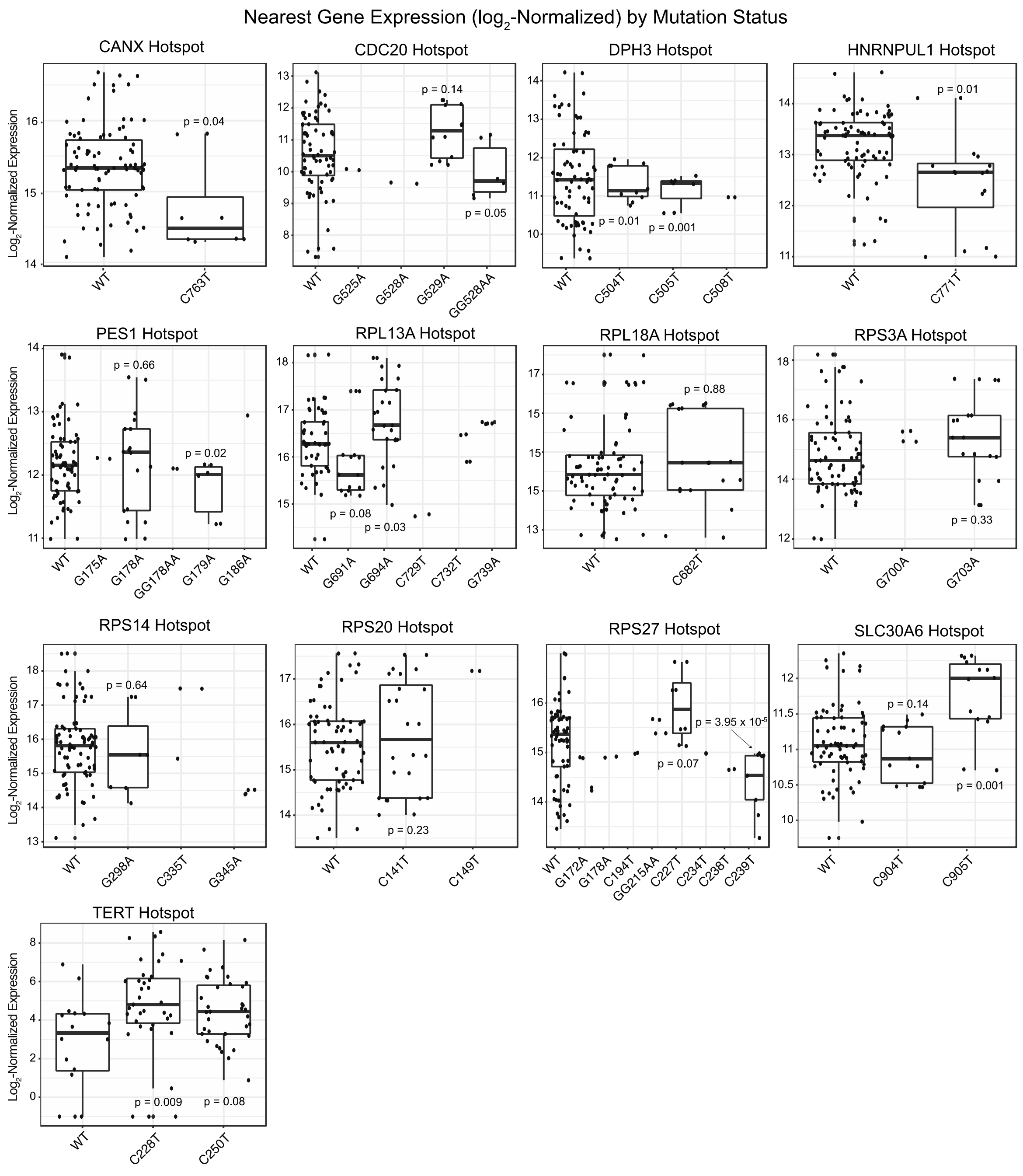

Supplemental Figure 2. Gene expression of nearest gene between WT and mutated samples. Boxplots of log_2-transformed DESeq2-normalized read counts from the ICGC-MELA RNA-sequencing cohort stratified by WT or mutation status. P-values were calculated using Student’s t-test.

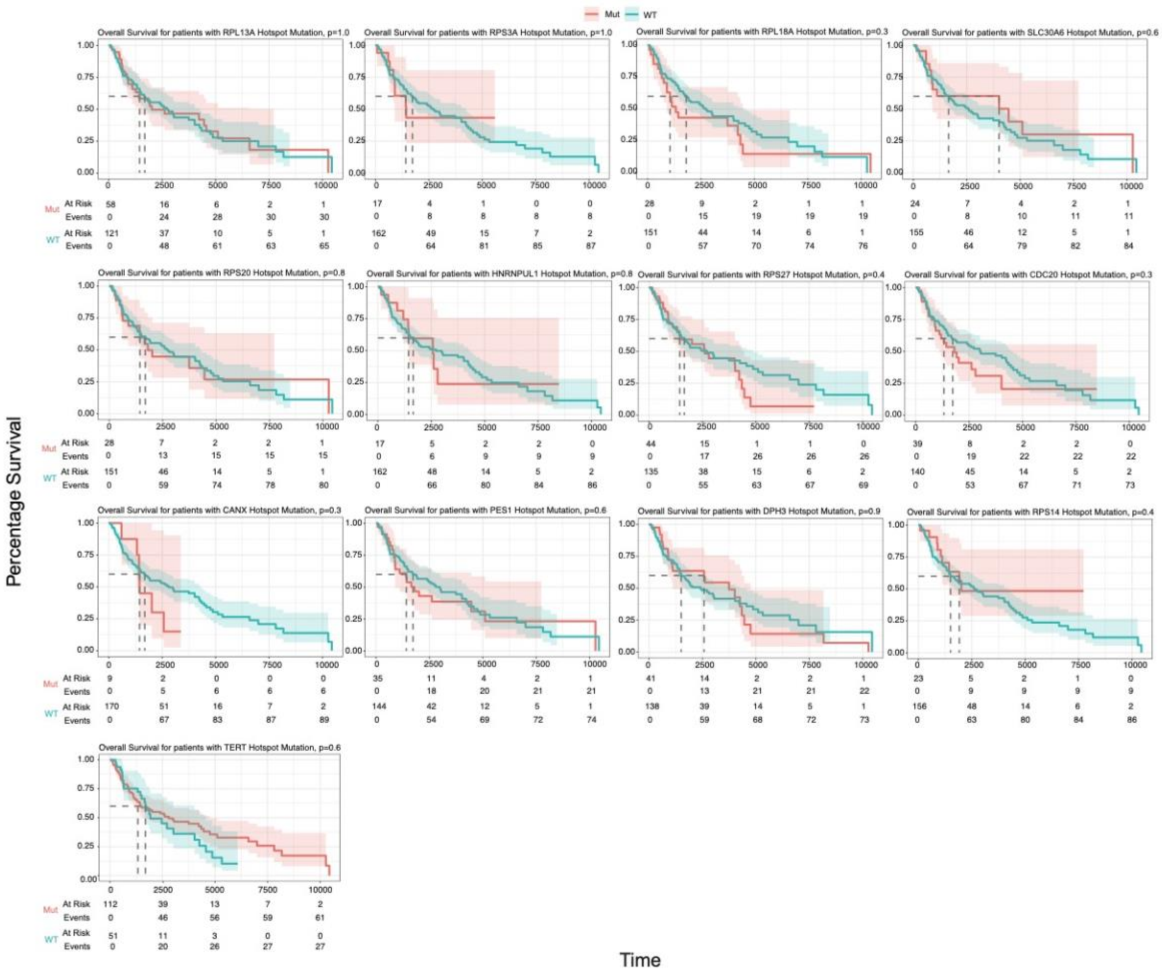

Supplemental Figure 3. Overall survival between wild-type and mutated samples for top 13 hotspot associated genes from TCGA. Kaplan-Meier curves depicting overall survival between wild-type (blue) and mutant (red) tumors. Numbers below each plot depict total number of patients with an event or at risk for each of the time points. Blue or red shaded regions represent the confidence intervals. Grey lines denote the timepoint at 60% survival. Plots are ordered from top left to bottom right in order of hotspot score.

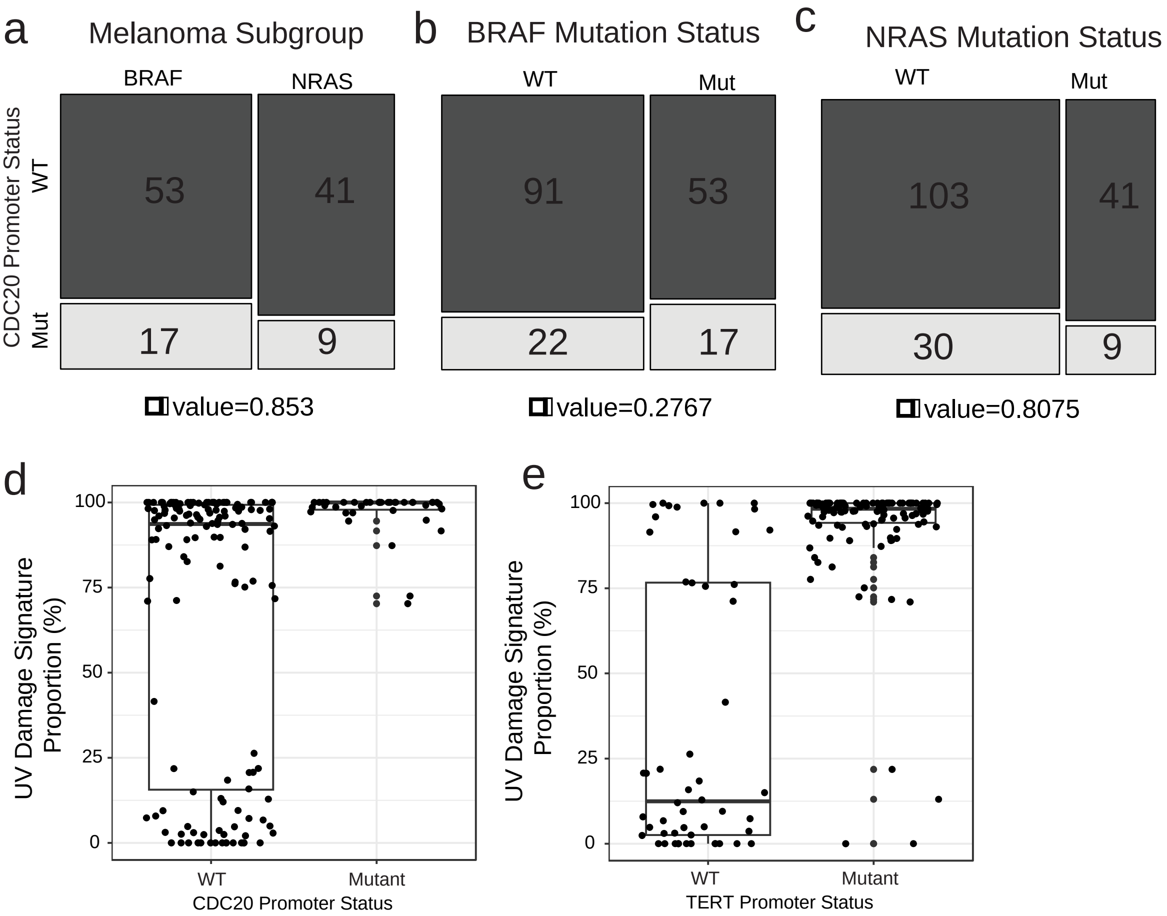

Supplemental Figure 4. Associations between the CDC20 promoter hotspot and clinical attributes. (a-c) Mosaic plot of the number of donors with WT and/or mutant CDC20 promoter and either **(a)** BRAF or NRAS mutations, **(b)** WT BRAF or mutant BRAF, **(c)** WT NRAS or mutant NRAS. **(d-e)** Boxplot depicting the proportion of UV signature per sample in the ICGC-MELA cohort with **(d)** a WT or mutated CDC20 promoter and **(e)** a WT or mutated TERT promoter.

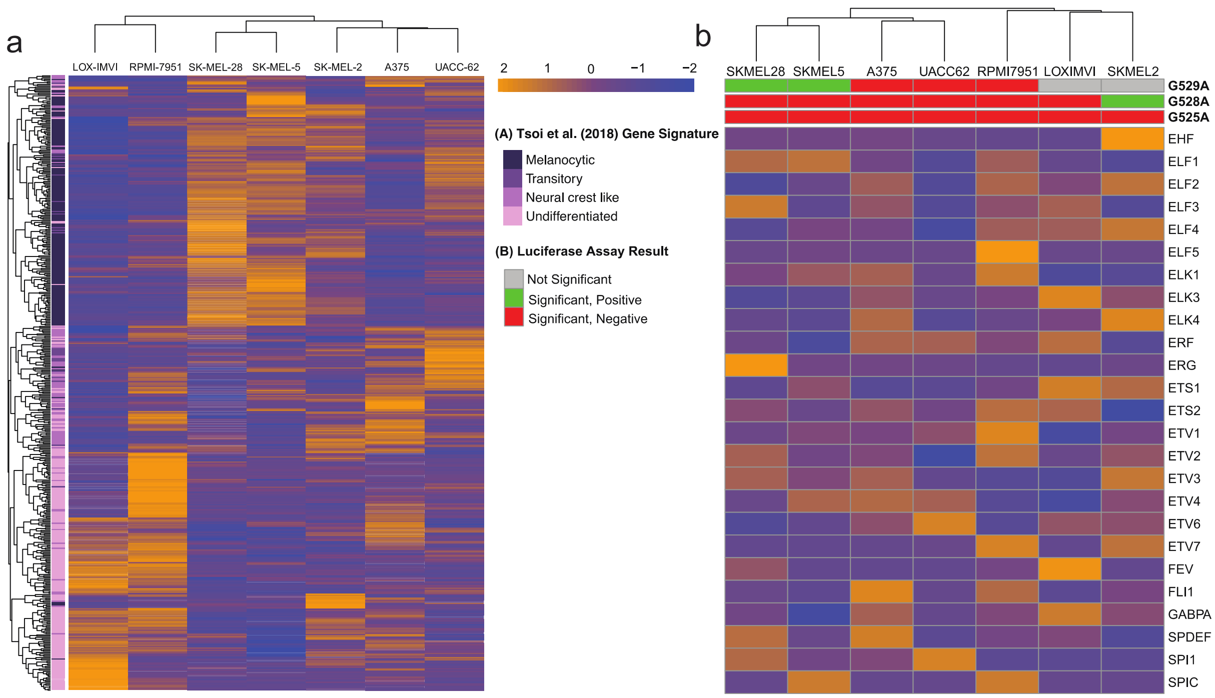

Supplemental Figure 5. Transcriptional heterogeneity in melanoma cell lines. (a) Unsupervised hierarchical clustering using the four major gene signatures generated by Tsoi et al. (2018). DESeq2-normalized reads were further normalized by z-score such that orange-colored cells indicate higher expression relative to other samples for the same gene. Genes represented by lighter purple colors belong to the undifferentiated or neural crest-like gene signature while darker purple colors represent genes in the melanocytic or transitory signature. **(b)** Unsupervised hierarchical clustering using all ETS transcription factor family members with non-zero expression. Values are normalized as in **(a).** The horizontal color bar indicates the result of the luciferase assay for the three most common CDC20 promoter variants. Grey indicates a non-significant change of the mutation compared to WT. Green indicates the mutation demonstrated higher reporter activity compared to WT. Red indicates that the mutation demonstrated lower reporter activity compared to WT.

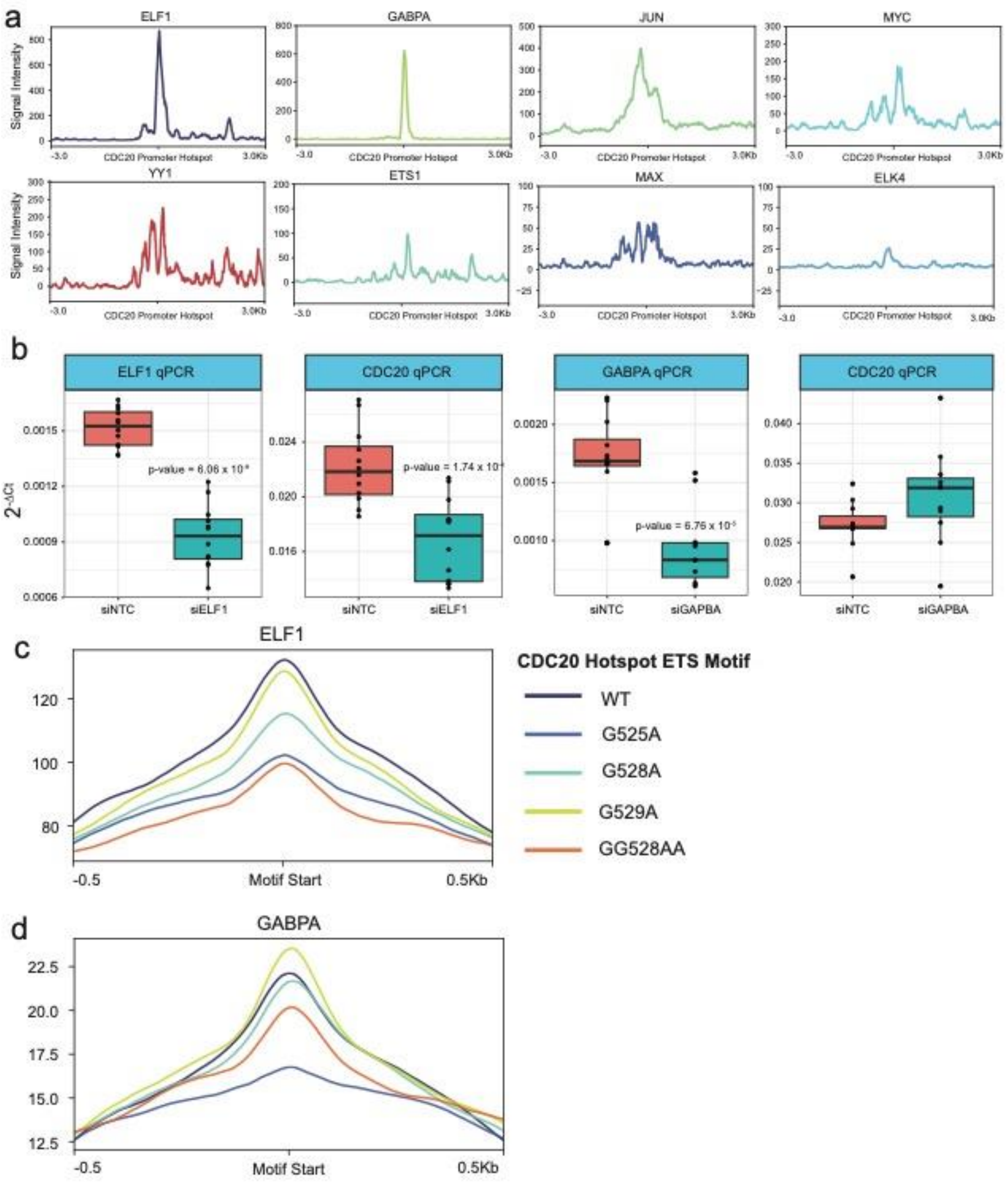

Supplemental Figure 6. Transcription factor activity at the CDC20 promoter hotspot. (a) Signal profile plots generated by deepTools. Each plot represents cumulative signal activity as assayed by ChIP-seq across multiple technical replicates for each transcription factor using publicly available ENCODE data from multiple cell types. All plots are centered at the start of the

36 bp CDC20 promoter hotspot and all activity included in the 3 kilo-bases flanking the hotspot are included in each hotspot. The transcriptional start site of CDC20 is 104 bp from the start of the CDC20 promoter hotspot. **(b)** Normalized delta-Ct scores obtained from qPCR of *ELF1*, *CDC20*, and *GABPA* in the presence of either siELF1 or siGABPA in A375 melanoma cell line, as indicated by the labels on the x-axis. NTC = non-targeting control. Each boxplot is made up from 12 points indicating separate technical replicates. **(c and d)** Signal profile plots using the same ChIP-seq bigWig input as in **(a)** but centered at 50,000 motifs within promoters in pMRRs that mimic the CDC20 promoter hotspot for **(c)** ELF1 and **(d)** GABPA.

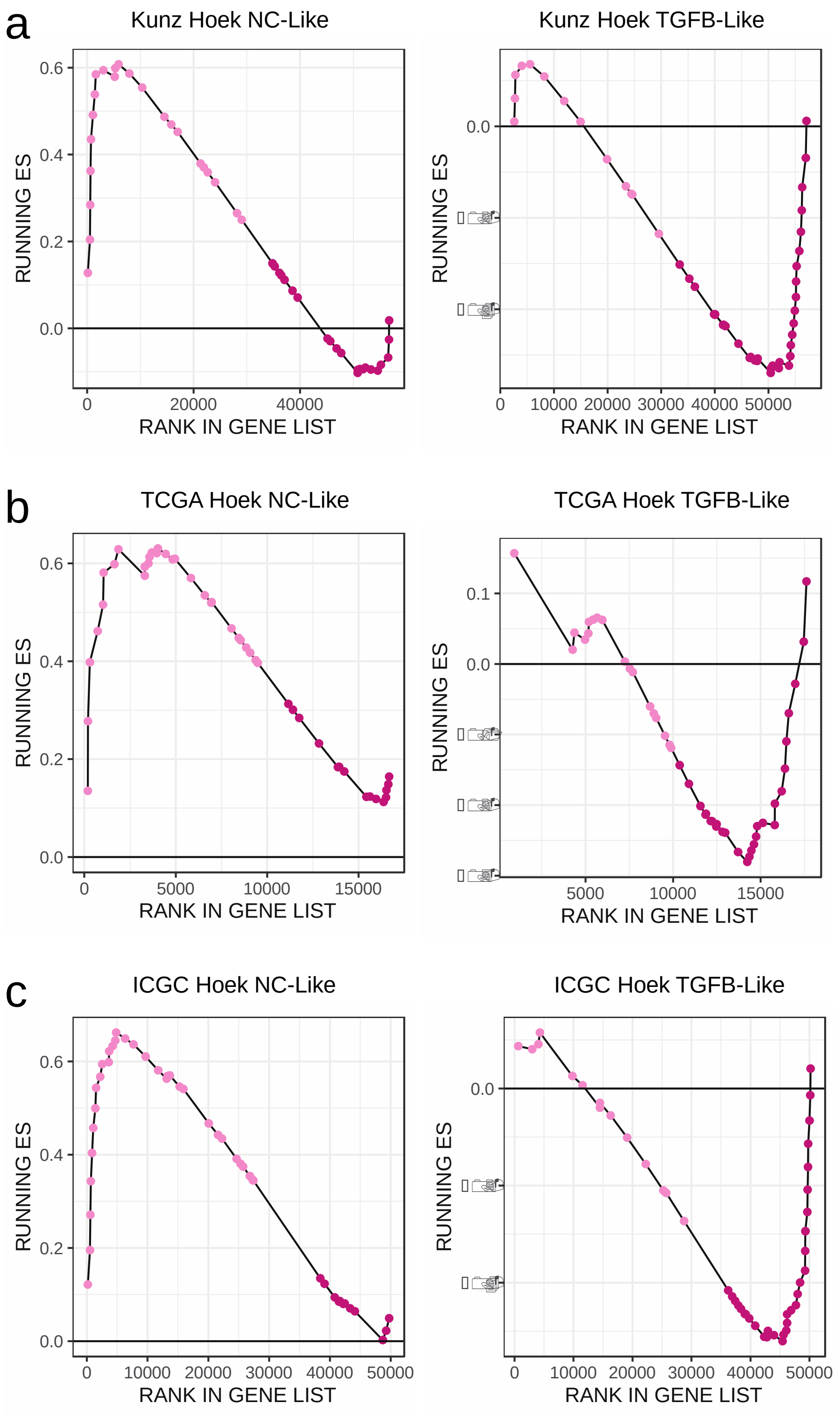

Supplemental Figure 7. Gene set enrichment analysis of CDC20-low and CDC20-high samples in three independent RNA-sequencing cohorts. GSEA results for the Hoek neural crest-like and TGFb-like signatures using CDC20-low (relative CDC20 expression less than the

25th quantile, light pink dots) and CDC20-high (relative CDC20 expression greater than the 75th quantile, darker pink dots) in the **(a)** Kunz cohort of primary melanomas and nevi, **(b)** TCGA-SKCM, **(c)** and ICGC-MELA.

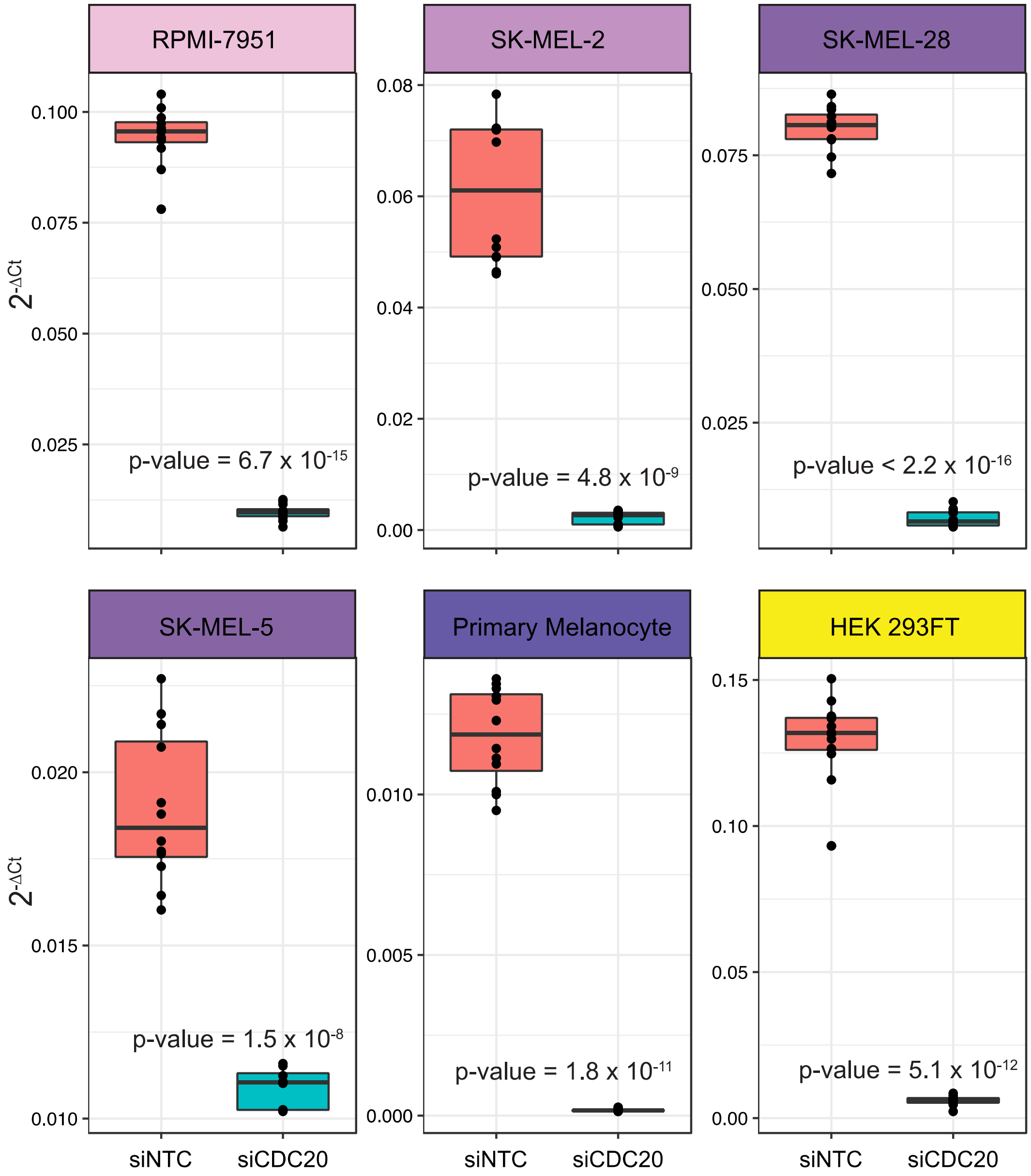

Supplemental Figure 8. Validation of *CDC20* knockdown by qPCR. Normalized Ct values for *CDC20* are plotted in cells transfected with a non-targeting siRNA control or with siCDC20. P-values were calculated using Student's t-test.

a

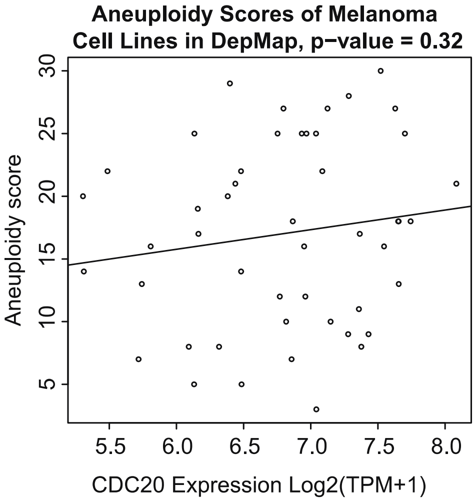

b

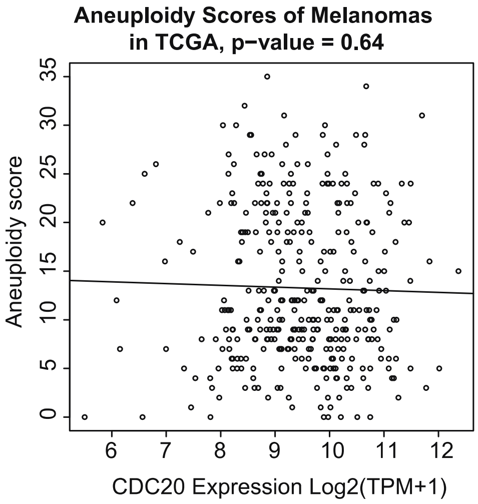

c

SNP ID	Karyotype/Nomenclature
A375	61~63<3N>,XX,-X/Y,+1,del(1)(p22p21),der(1)t(1;6)(p13,p12),-2,add(2)(q22),-4,-6,der(6) t(6;22)(q10;p10),der(8)(t(4;8)p10;q10),-10,del(11)(p15),del(13)(q33), add(15)(p11.2),del(17) (p11.2),-18,add(19)(p13),-21, -22x2,+2~4 mar[cp20]
A3	60~63<3N>,XX,-X/Y,+1,del(1)(p22p21),der(1)t(1;6)(p13,p12),-2,add(2)(q22),-4,-6,der(6)t(6;22)(q10;p10),der(8)t(4;8)(p10;q10),-10,del(11)(p15),-13, del(13)(q33), add(15) (p11.2),-18,add(19)(p13),-21,-22x2,+1~2 mar[cp20]
A10	61~63<3N>,XX,-X/Y,+1,del(1)(p22p21),der(1)t(1;6)(p13,p12),-2,add(2)(q22),-4,-6,der(6)t(1;6) (p34;p12),der(8)(t(4;8)p10;q10),-10,del(11)(p15),add(13)(p11.2), del(13)(q33), -18,add(19) (p13),-21,-22x2,+2~3 mar[cp20]

d

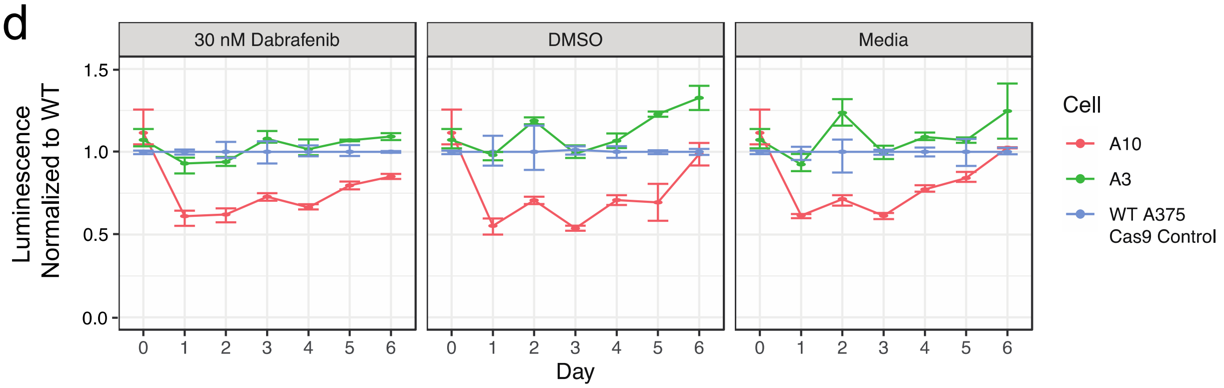

Supplemental Figure 9. Aneuploidy and viability of WT and CDC20 promoter indel cell lines. (a-b) Association between normalized *CDC20* expression and aneuploidy score in **(a)** melanoma cell lines in the DepMap cohort and **(b)** the TCGA-SKCM cohort. **(c)** Table showing the karyotype/nomenclature of WT A375, A3, and A10. **(d)** Proliferation rates are slightly lower in A10 but unchanged in A3. Plot shows luminescence values obtained from CellTiterGlo normalized to the average WT luminescence for each day and for each specific condition. Each point represents the average of three replicates. Confidence intervals are calculated using a nonparametric bootstrap method.
