# Supplementary material for: Functional analysis of recurrent CDC20 promoter variants in human melanoma
Source: Commun Biol. 2023 Nov 29;6:1216. doi: 10.1038/s42003-023-05526-2 (PMC10686982; doi:10.1038/s42003-023-05526-2)
Supplement: Supplementary file 3 — Description of Additional Supplementary Files [file 42003_2023_5526_MOESM3_ESM.pdf]

Description of Additional Supplementary Files

File name: Supplemental Data 1
Description: Datasets used to annotate putative melanoma regulatory regions.

File name: Supplemental Data 2
Description: List of statistically significant hotspots

File name: Supplemental Data 3
Description: Source Data for Figure 1

File name: Supplemental Data 4
Description: P-values and fold changes from luciferase assay.

File name: Supplemental Data 5
Description: Source Data for Figure 2

File name: Supplemental Data 6
Description: ENCODE datasets used to analyze transcription factor binding activity at CDC20 promoter.

File name: Supplemental Data 7
Description: GSEA results using the Hoek gene signature.

File name: Supplemental Data 8
Description: Source Data for Figure 3b-e

File name: Supplemental Data 9
Description: RNA-sequencing results for WT, A3, and A10.

File name: Supplemental Data 10
Description: Source Data for Fig. 5a and 5c

File name: Supplemental Data 11
Description: Results from in vivo tumor growth curve.

File name: Supplemental Data 12
Description: List of primers and reagents.
